# Investigating the Marginal and Herd Effects of COVID-19 Vaccination for Reducing Case Fatality Rate: Evidence from the United States between March 2021 to January 2022

**DOI:** 10.3390/vaccines11061078

**Published:** 2023-06-09

**Authors:** Tenglong Li, Zilong Wang, Shuyue He, Ying Chen

**Affiliations:** 1 Wisdom Lake Academy of Pharmacy, Xi’an Jiaotong-Liverpool University, Suzhou 215123, China; 2 Department of Financial and Actuarial Mathematics, Xi’an Jiaotong-Liverpool University, Suzhou 215123, China

**Keywords:** COVID-19 vaccine, herd effect, marginal effect, case fatality rate, segmented regression

## Abstract

Vaccination campaigns have been rolled out in most countries to increase vaccination coverage and protect against case mortality during the ongoing pandemic. To evaluate the effectiveness of COVID-19 vaccination, it is vital to disentangle the herd effect from the marginal effect and parameterize them separately in a model. To demonstrate this, we study the relationship between the COVID-19 vaccination coverage and case fatality rate (CFR) based on U.S. vaccination coverage at county level, with daily records from 11 March 2021 to 26 January 2022 for 3109 U.S. counties. Using segmented regression, we discovered three breakpoints of the vaccination coverage, at which herd effects could potentially exist. Controlling for county heterogeneity, we found the size of the marginal effect was not constant but actually increased as the vaccination coverage increased, and only the herd effect at the first breakpoint to be statistically significant, which implied an indirect benefit of vaccination may exist at the early stage of a vaccination campaign. Our results demonstrated that public-health researchers should carefully differentiate and quantify the herd and marginal effects when analyzing vaccination data, to better inform vaccination-campaign strategies as well as evaluate vaccination effectiveness.

## 1. Introduction

The world has been living in the shadow of COVID-19 pandemic since the outbreak in 2019, and COVID-19 posed unprecedented challenges to healthcare systems and resulted in high mortality during the initial stage, due to the difficulties in identifying efficient and effective cures [1] Great hope has been placed in COVID-19 vaccines to end the pandemic, as clinical-trial results suggested COVID-19 vaccination can effectively prevent symptomatic infections, especially severe symptoms, which protects against mortality associated with infections [2,3,4]. For this reason, public demand for COVID-19 vaccines was fervent and vaccination campaigns were initiated all over the world, for an early safe vaccine supply for populations at risk as well as a massive vaccine supply to match the public’s demand [5,6]. For example, the Food and Drug Administration (FDA) issued emergency-use authorizations (EUA) for the Pfizer-BioNTech and Moderna COVID-19 vaccines in December 2020, which marked the beginning of the vaccination campaign in the U.S. COVID-19 vaccines were then first allocated to populations at risk, the elderly population (age 65+) and frontline (mostly healthcare and education) workers. After President Biden announced that all Americans would be eligible for COVID-19 vaccines by 1 May 2021, the vaccination campaign was further accelerated [7]. Booster doses of COVID-19 vaccines were introduced to restore the level of protection (antibody) which eroded over time [8,9,10]. By 24 November 2022, more than 80% of Americans have received at least one dose and more than 68% of Americans have completed a primary series of COVID-19 vaccine [11]. The literature has reported that the vaccination coverage is negatively associated with case fatality rate (CFR), which refers to the mortality rate among those who are infected (i.e., confirmed COVID-19 cases) [12,13].

It is necessary to decompose the protection effect of COVID-19 vaccines against case mortality, to better understand the underlying mechanism [14]. The protection effect of COVID-19 vaccines is, in general, a mix of two different effects, i.e., the direct effect and the indirect effect [15]. The direct effect refers to direct protection of inoculated individuals against case mortality, as the vaccines can effectively reduce individual susceptibility to COVID-19 infection and severe symptoms [14,15]. The indirect effect, however, is a bit abstract and potentially related to herd immunity, which is a conception which states that transmission of the SARS-CoV-2 viruses can be largely prevented if a fixed proportion of the population is immunized (either by vaccination or by recovery from infection; this proportion is called the herd-immunity threshold), rendering COVID-19 insignificantly dangerous for public health as its basic reproduction number goes below one [16,17]. The indirect effect is defined as the protection gained by unvaccinated people against case mortality, as the vaccination coverage in the population increases, potentially because the vaccination can reduce both the number and the infectiousness of infected people in the population, which works following a mechanism very similar to herd immunity [14]. The above concepts of the direct and indirect effects can be contextualized within the investigation of the impact of COVID-19 vaccination on case fatality rate (CFR). The direct effect could be interpreted as the reduction in CFR associated with one unit/percent increase in the vaccination coverage, i.e., the direct effect evaluates the marginal gain during a vaccination campaign. For this reason, the direct effect is referred to as the marginal effect (for reducing the CFR) in this paper. The indirect effect could be interpreted as the additional reduction in CFR when the vaccination coverage reaches certain unknown levels, i.e., the indirect effect quantifies potential sudden drops in CFR at certain unknown locations during the process of vaccinating a target population. To better characterize its nature, the indirect effect is referred to as the herd effect (for reducing the CFR) in this paper.

It is particularly important to distinguish the herd effect from the herd immunity. First, the herd effect is defined for (reducing) mortality while the herd immunity is defined for (reducing) infections, and therefore they are two concepts with distinct natures. Undoubtedly, any herd effect potentially depends on the herd immunity as mortality is caused by infections. Second, those two concepts have very different implications for modeling and calculations: The herd-immunity threshold has an explicit theoretical model and can be easily calculated based on the reproduction number, while the herd effect has no known/theoretical model except that it is related to the vaccination coverage, mostly because mortality results from a much more complex mechanism compared to infections. To summarize, the herd-immunity threshold is a one single threshold that can be calculated in a straightforward manner [18], while the herd effect would (at least) require a regression model and may be discovered at different unknown locations (different levels of vaccination coverage) for reducing the CFR [19]. Consequently, estimating the herd effect typically involves two steps: (1) estimating/identifying the locations (levels of vaccination coverage) where the herd effect can be found; and (2) estimating the sizes of the herd effect at the identified locations.

Our primary goal in this paper is to disentangle the herd effect from the marginal effect, for the following three reasons: First, the marginal and herd effects address different scientific questions with regard to distinct groups of people (i.e., the vaccinated individuals versus the unvaccinated individuals). Second, as discussed earlier, the herd effects may be discovered at different unknown locations, and those locations essentially delineate different stages in a vaccination campaign where the marginal and herd effects may not be constant across those stages. Third, given the aforementioned two reasons, a deeper knowledge about the protection effect of vaccination is likely gained by establishing the marginal and herd effects, and vaccination strategies could be optimized for a target population based on such knowledge. Investigation and discussion about the herd and marginal effects have been neglected thus far in the open literature. To achieve the above goal, we estimate the herd and marginal effects based on a dataset from the U.S. Centers for Disease Control and Prevention (CDC), which records various vaccination coverages for each U.S. county daily [11]. We hypothesize that both the herd and marginal effects exist and are significantly negative for modeling CFR. The segmented regression is employed first to identify the breakpoints which are considered the thresholds for triggering the herd effect, based on data of all the U.S. counties included in our study. With the identified breakpoints, we estimate the herd and marginal effects at national level using segmented regression and at county level using a mixed model. Data on the social vulnerability index (SVI) for individual counties is also included to control for health disparities due to sociodemographic factors at county level [20]. Heterogeneity among individual counties is further evaluated by the random effects associated with the herd and marginal effects in a mixed model.

## 2. Materials and Methods

### 2.1. Data

We conducted an ecological study which was built on data from three different sources. The US vaccine administration and equity dataset was obtained from the CDC website and has vaccination coverages of the general population and its subpopulations (defined by age) recorded daily at county level [11]. The percentage of people who completed a primary series of vaccination in the general population was extracted from the dataset and served as the main covariate in our model. The daily CFR at county level was calculated as the ratio of the daily count of deaths and the daily count of COVID-19 cases, based on the time-series summary tables of COVID-19 deaths and confirmed cases, which were accessed from the COVID-19 data repository by the Center for Systems Science and Engineering (CSSE) at Johns Hopkins University [21,22]. To further control for county heterogeneity, we used a dataset from the Centers for Disease Control and Prevention Social Vulnerability Index (CDC SVI) database, created by the Geospatial research, Analysis & Services Program under the Agency for Toxic Substances and Disease Registry [20]. The CDC SVI database was established to help health officials and emergency-response planners identify counties that will most likely need support before, during, and after a hazardous event. CDC SVI ranks counties on 15 social factors and further groups them into four themes, namely, socioeconomic status, household composition and disability, minority status and language, and housing type and transportation [23]. We chose to use the theme-specific ranking which was constructed by summing the percentiles of the factors under each theme. The theme-specific ranking was set in the range from 0 to 1, with higher values indicating greater vulnerability.

The vaccination coverages and daily CFR for the period between 11 March 2021 and 26 January 2022 were selected. We chose 11 March 2021 as it was the date when President Biden announced that COVID-19 vaccine would be available for all American adults by 1 May 2021, an event which marked the beginning of massive vaccination campaign in the U.S. We chose 26 January 2022 as the end date of our study as it was reported on this date that Omicron variant accounted for 99.9% of the new infections. This would alleviate the concern of potential confounding effect of Omicron variant regarding the relationship between the vaccination coverage and CFR. Thirty-one counties with missing values on county FIPS code, vaccination coverages, the CDC SVI or CFR were excluded, and the final dataset has 1,001,098 observations clustered by 3109 U.S. counties. To prepare the dataset for analysis at national level, we further extracted the average CFR and average vaccination coverage (i.e., the percent of people who completed a primary series of COVID-19 vaccine) across all the counties in our dataset for each day during our study period.

### 2.2. Models

Segmented regression models were employed to estimate the herd and marginal effects. Segmented regression is very similar to ordinary regression, with the only difference that regression coefficients should be estimated repeatedly for different local regions whose boundaries are defined by breakpoints, which represent the locations of structural changes in regression models [24,25]. Typically, the first step is to determine the number of breakpoints, which can be achieved by a model-selection-like procedure, i.e., models with different numbers of breakpoints are compared in terms of their model-fit indices (such as AIC or BIC) to determine the optimized number of breakpoints. The second step is to estimate the locations of breakpoints given the number of breakpoints. The third step, based on the estimated breakpoints, is then to fit regression models to different local regions separated by the breakpoints. Normally, one would expect all regression coefficients to be changeable across different regions, unless otherwise specified.

For our analysis at national level, we intended to examine the relationship between CFR and the vaccine coverage, based on the dataset comprised of only the average vaccination coverage and CFR in the U.S. The following regression model was formulated for the analysis at national level:

(1) $$y_{t}=\beta_{0}+\beta_{1}X_{t}+\sum_{k=1}^{m}\alpha_{k}I_{\left\{X_{t}\in \Psi_{k}\right\}}+\sum_{k=1}^{m}\delta_{k}{X_{t}I}_{\left\{X_{t}\in \Psi_{k}\right\}}+\varepsilon_{t}$$

where $y_{t}$ and $X_{t}$ denote the average CFR and vaccinate coverage in the U.S. on day *t*. Model (1) is built on the estimated breakpoints $b_{1}<b_{2}<\cdots <b_{m}$, which implies there are m+1 different local regions and m different breakpoints in total (except $b_{0}$ and $b_{m+1}$ which are the minimum and maximum of $X_{t}$). The local regions separated by the breakpoints are denoted by $\Psi_{k}=\left[b_{k},b_{k+1}\right)$ for k=1,2,⋯,m. The reference region $\Psi_{0}$, although omitted from Model (1), refers to the local region $\Psi_{0}=\left[b_{0},b_{1}\right)$. The indicator function $I_{\left\{X_{t}\in \Psi_{k}\right\}}$ creates the dummy variable which assigns value 1 if the value of $X_{t}$ falls in the local region $\Psi_{k}$ and 0 otherwise, which operationally divides the range of $X_{t}$ into the local regions. The marginal effects in those local regions are characterized by $\beta_{1}$ for the reference region and $\beta_{1}+\delta_{k}$ for the local region $\Psi_{k}$, and these parameters can be interpreted as the marginal gain/drop in CFR if the vaccination coverage increases by one percent. The herd effects at the breakpoint $b_{k}$ are characterized by $\alpha_{k}\;-$ $\alpha_{k-1}$ (for the breakpoint $b_{1}$ it is just $\alpha_{1}$), and the herd effect can be interpreted as the additional gain/drop in CFR if the vaccination coverage passes its corresponding breakpoint/threshold.

The breakpoints $b_{k}$, k=1,2,⋯,m are estimated based on Model (1) and the dataset for the analysis at national level (i.e., with only average daily CFR and vaccination rate in the U.S.). Naturally, they reflect the structural changes in the relationship between CFR and the vaccination coverage in general, and they can be applied to the analysis at county level where we used the longitudinal data (322 days) for all the counties (3109 counties), along with the CDC SVI indicators for explaining county heterogeneity. We built the following mixed model for the analysis at county level:

(2) $$y_{it}=\beta_{0}+\beta_{1}X_{it}+\sum_{k=1}^{m}\alpha_{k}I_{\left\{X_{it}\in \Psi_{m}\right\}}+\sum_{k=1}^{m}\delta_{k}{X_{it}I}_{\left\{X_{it}\in \Psi_{m}\right\}}+\gamma Z_{i}+u_{i1}X_{it}+u_{i0}+\sum_{k=1}^{m}{\tilde{\alpha }}_{ik}I_{\left\{X_{it}\in \Psi_{m}\right\}}\;+\sum_{k=1}^{m}{\tilde{\delta }}_{ik}{X_{it}I}_{\left\{X_{it}\in \Psi_{m}\right\}}+\varepsilon_{it}$$

where $y_{it}$ and $X_{it}$ denote the CFR and vaccination coverage for county *i* at day *t*. $Z_{i}$ is the covariate vector which contains CDC SVI theme-specific rankings on the four main themes for county *i*. $\beta_{0},\beta_{1},\alpha_{k},\delta_{k}$ are the parameters characterize the herd and marginal effects, as similarly defined in Model (1), except in Model (2) they are fixed effects. Correspondingly, we have their random effects characterized by $u_{i0},u_{i1},{\tilde{\alpha }}_{ik},{\tilde{\delta }}_{ik}$ for an individual county *i*, which is due to the heterogeneity among the counties that cannot be explained away by the fixed effects of county rankings on CDC SVI (which are represented by γ). In addition, we use G to represent the variance–covariance matrix of the random effects $u_{i0},u_{i1},{\tilde{\alpha }}_{ik},{\tilde{\delta }}_{ik}$, and the variances of the random effects (VRE) are used to the variations of the sizes of the herd and marginal effects across individual counties. For example, the fixed effect $\alpha_{1}$ characterizes the size of first herd effect for the general population of the counties in our study, while its random effect ${\tilde{\alpha }}_{i1}$ characterizes the size of first herd effect for the individual county *i* specifically. Therefore, the variance of ${\tilde{\alpha }}_{i1}$ naturally quantifies the variation of the size of the first herd effect across all individual counties. Model (2) is built on the same set of breakpoints $b_{k}$, k=1,2,⋯,m, which were obtained based on Model (1) and the dataset for the analysis at national level. This means Model (2) shares the same local regions $\Psi_{k}=\left[b_{k},b_{k+1}\right)$ for k=1,2,⋯,m, across all the counties in our study. The significances of the fixed effects $\beta_{0},\beta_{1},\alpha_{k},\delta_{k}$ as well as their corresponding random effects $u_{i0},u_{i1},{\tilde{\alpha }}_{ik},{\tilde{\delta }}_{ik}$ will be checked via model outputs and comparison tests. Model (2) was fitted to the data using the STATA (version 17.0) software and the maximum likelihood estimation method (see Supplementary Materials for details).

## 3. Results

### 3.1. The Results of the Analysis at National Level

**Figure 1:**
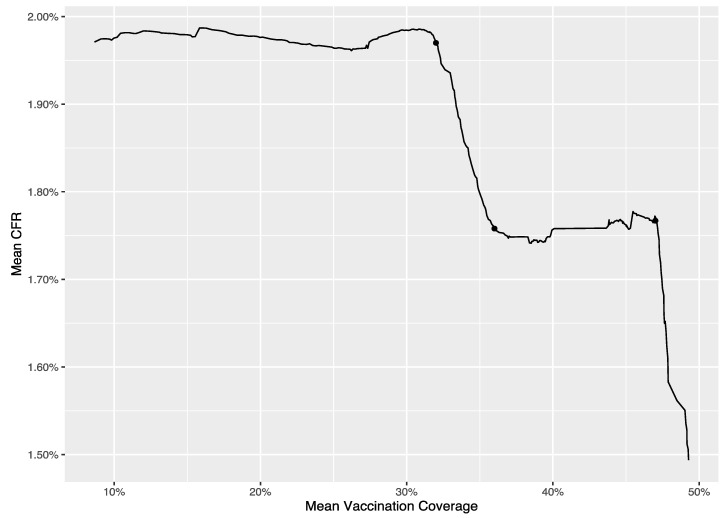
The relationship between the average vaccination coverage and the average CFR in the U.S. The solid dots represent the breakpoints estimated by the “segmented” package in R.

As mentioned above, the dataset used for the analysis at national level has two variables, i.e., average daily CFR and average vaccination coverage in the USA. The breakpoints were estimated based on this dataset using the “segmented” package in R (version 4.2.0) [25]. To avoid overfitting, we set the maximum number of the breakpoints as 3, based on the curve between the average daily CFR and the average daily vaccination coverage depicted in Figure 1. The segmented package then performed an automatic selection of the number of breakpoints based on BIC, and it estimated the locations of the breakpoints conditional on the optimized number of the breakpoints. The estimated breakpoints were superimposed on the curve in Figure 1, to further validate those estimates align with the observed structural changes.

The breakpoints were estimated as 32%, 36% and 47%, which suggested that the herd effect for reducing CFR (protecting against mortality) may be found when the vaccination coverage reached 32%, 36% and 47%. Based on those breakpoints, we have four different local regions, namely, $\Psi_{1}=\left[8.66\%,32\%\right);\Psi_{2}=\left[32\%,36\%\right);\Psi_{3}=\left[36\%,47\%\right);\Psi_{4}=\left[47\%,49.28\%\right)$, with the minimum and maximum of the average daily vaccination coverages as 8.66% and 49.28%, respectively. Table 1 lists the estimates of the regression coefficients based on Model (1). We further calculated the herd- and marginal-effect estimates, which are tabulated in Table 2. The marginal effect in the first local region $\Psi_{1}$ was insignificant, which suggested that the drop in the CFR per percent increase in the vaccination coverage was not significantly different from 0, if the vaccination coverage did not surpass 32%. The herd effect at the breakpoint 32% was also insignificant, which was largely due to the insignificant marginal effect in the region $\Psi_{1}$. We found a significant marginal effect in the second local region $\Psi_{2}$ (−0.057), which indicated that there was a drop by 0.057 percent in the CFR for every percent increase in the vaccination coverage in this region, evidencing the protection effect of COVID-19 vaccination against mortality. In addition, the herd effect at the breakpoint 36% was significant too (−0.233), suggesting that there was a further drop by 0.233 percent in the CFR besides the marginal CFR reduction per percent increase in the vaccination coverage. In the third local region $\Psi_{3}$, however, we observed a slight positive marginal effect in the CFR (0.003), which means the marginal gain of vaccination (in terms of the reduction in CFR) disappeared and vaccination was somehow harmful to protecting against mortality. Correspondingly, the herd effect at the breakpoint 47% was also positively significant (0.009), suggesting again that vaccination was not helpful at this stage. The marginal effect in the fourth local region $\Psi_{4}$ was strongly negative; specifically, there was a drop by 0.115 percent in the CFR associated with every percent increase in the vaccination coverage at this stage.

### 3.2. The Results of the Analysis at County Level

We further investigated the marginal and herd effects of COVID-19 vaccination based on an analysis at county level, where the daily CFR and vaccination coverages from 11 March 2021 to 26 January 2022 as well as the CDC SVI rankings for 3109 U.S. counties were used. The estimated breakpoints of 32%, 36% and 47%, obtained based on the analysis at national level, were adopted for our analysis at county level. The mixed Model (2) was employed to account for the clustered data at county level, and its fixed- and random-effect estimates are tabulated in the Table 3. Furthermore, the estimates of herd and marginal effects, as well as their corresponding random-effect estimates, are listed in Table 4. To determine the significance of the random effects, we compared the full model (i.e., Model (2)) with two different reduced models (one without the random effects associated with all the marginal effects, i.e., $u_{i1},{\tilde{\delta }}_{i1},{\tilde{\delta }}_{i2},{\tilde{\delta }}_{i3}$ and another one without the random effect associated with the first marginal effect only, i.e., $u_{i1}$), and the resultant tests gave *p*-values smaller than 0.001, suggesting that it was necessary to include random effects for all the marginal- and herd-effect parameters.

Across all the U.S. counties in our data, the marginal effect was significantly negative in the first local region $\Psi_{1}$ (i.e., when the vaccination coverage was between 8.66% and 32%); specifically, a one percent increase in the vaccination coverage was associated with a 0.004 percent drop in the CFR. The first herd effect at the breakpoint of 32% was −0.025 and significant, meaning there was an additional drop by 0.025 percent in the CFR as the vaccination coverage reached 32%, beyond the marginal effect observed in $\Psi_{1}$. The marginal effect in the second local region $\Psi_{2}$ was also significantly negative (−0.01), which showed that there was a 0.01 percent drop in the CFR per one-percent increase in the vaccination coverage, when the vaccination coverage was between 32% and 36%. The second herd effect, however, was insignificant overall, which suggested the additional protection against mortality at the breakpoint 36% may not exist. Similarly, we found a significant marginal effect (−0.023) for the third local region $\Psi_{3}$ but an insignificant herd effect at the breakpoint 47%. The marginal effect within the fourth local region $\Psi_{4}$ was the strongest, as every percent increase in the vaccination coverage was associated with a 0.043 percent reduction in the CFR, if the vaccination coverage surpassed 47%.

Furthermore, heterogeneity among the U.S. counties regarding the herd- and marginal-effect estimates was evident. The distributions of those herd and marginal effects can be approximately obtained based on their fixed-effect estimates and the variance estimates of their random effects. For the first herd effect (at 32%), the fixed-effect estimate was −0.025 with the variance of its random effect as 0.024, and this means the first herd effect approximately followed a normal distribution whose mean was −0.025 and variance was 0.024 across individual counties. This indicates that roughly 56% of the counties had negative herd effects as expected, but the other 44% of the counties could have no herd effect or even positive herd effects at the breakpoint 32%. For the second and third herd effect (at 36% and 47%, respectively), roughly 49% of the counties have negative herd effects, which further demonstrated that those two herd effects were not significant among the counties. Regarding the marginal effects: although their fixed-effect estimates were all very significant (*p*-value < 0.001), their random-effect estimates suggested the fourth marginal effect was the strongest (it was negative in 73% of the counties). The first, second and third marginal effects were negative in approximately 57%, 54% and 62% of the U.S. counties. To summarize, the protection effect of COVID-19 vaccination against mortality was confirmed in general and for the majority of the U.S. counties, while substantial heterogeneity which defined the size and the validity of the protection effect for individual counties still existed. We also found that only one CDC SVI theme ranking, i.e., rankings on household composition and disability, could help explain the county heterogeneity. Unsurprisingly, this CDC SVI theme ranking was positively related to CFR, and, specifically, a one percentile rise in the theme ranking could result in a 0.8 percent increase in CFR.

## 4. Discussion

Vaccination has been acknowledged as an effective tool to reduce hospitalization and mortality related to COVID-19 infections, and vaccination campaigns have been rolled out in virtually every country that has access to COVID-19 vaccines. Understanding the effect of COVID-19 vaccination in terms of case-fatality-rate (CFR) reduction has an unquestionably profound meaning for the successful implementation of COVID-19 vaccination campaigns. Drawing on the direct and indirect effects of vaccination from the literature, we rename the direct effect as the marginal effect against mortality and the indirect effect as the herd effect against mortality, to better describe the nature of those effects in terms of reducing the CFR. Defining the herd and marginal effects also helps build regression models for obtaining their estimates, as those two kinds of effects require different parameterization in the model. Analysis at the national level and county level for the United States were then implemented based on datasets containing the daily vaccination coverages and case reports in the U.S. Theme rankings for individual counties from the CDC SVI were also included to explain heterogeneity at county level. Our analysis at national level suggested three different locations (i.e., when the vaccination rate reached 32%, 36% and 47%) for possible herd effects and strong significance for the marginal effects, which was further confirmed by our analysis at county level after controlling for county heterogeneity.

Our analyses demonstrated how COVID-19 vaccination protects against COVID-19-related mortality over the course of the COVID-19 vaccination campaign in the U.S. In general, COVID-19 vaccination can indeed significantly reduce the CFR, but its effect was not constant during the vaccination campaign. The estimated breakpoints divided the vaccination campaign into four different regions based on the vaccination coverage, i.e., $\Psi_{1}=\left[8.66\%,32\%\right)$, $\Psi_{2}=\left[32\%,36\%\right)$, $\Psi_{3}=\left[36\%,47\%\right)$ and $\Psi_{4}=\left[47\%,49.3\%\right)$. The marginal effects in those four regions are, correspondingly, −0.004, −0.01, −0.023 and −0.043, which are all significant. This shows that vaccination can directly result in a meaningful reduction in the CFR and, thus, it should be recommended especially for the unvaccinated population, as the marginal effects largely quantify the reduced risks of mortality that one would benefit from the vaccination if he/she chooses to get vaccinated. We also observe that the sizes of the marginal effects increase as the vaccination coverages increases, which suggests that the direct benefit of COVID-19 vaccination against mortality becomes more and more significant as the vaccination coverage in the population increases. Our results also indicate the existence of a herd effect, specifically at the location 32%. The herd effect at the location 32% is statistically significant (−0.025), which demonstrates the indirect (additional) protection against mortality brought by the vaccination if the vaccination coverage reaches 32% in the population. This implies that one would indirectly benefit from the COVID-19 vaccination even if he/she is not vaccinated as long as the vaccination coverage passes 32%, by a 0.025% reduction in the CFR. One possible explanation for this low threshold of herd effect is that the at-risk populations were actually vaccinated first in the U.S., which averted a significant number of deaths and hospitalizations (that would have occurred without the vaccination campaign) [26,27,28].

Our results lead to some practical implications for COVID-19 vaccination strategies. First, our findings suggest that the herd effects for reducing COVID-19 mortality may exist at the early stage of a vaccination campaign. This implies early vaccination may still effectively protect high-risk populations. However, our findings do not naturally support a recommendation of a massive COVID-19 vaccination, which may not be cost-effective and could be undermined by the dominance of Omicron. This echoes our earlier finding of the significant herd effect at the first breakpoint of 32% and is consistent with recommendations offered by the literature [4,5,26,29,30]. It is noteworthy that a rapid, effective implementation at the initial stage can pose considerable logistical challenges for a vaccination campaign [29,31,32]. Therefore, careful resource planning is required for the access to, transportation, storage and distribution of vaccines, which has been exemplified by the vaccination campaign in the U.S. [26,33]. Second, eligible unvaccinated individuals should be encouraged to get vaccinated at all stages of a vaccination campaign, as the marginal effects were evident across all the local regions defined for the U.S. vaccination campaign in our analysis. More profoundly, we found that the whole population would benefit more if more people got vaccinated, as the size of marginal effect was positively correlated with the vaccination coverage in the population. The gain from the marginal effects, on average, also outweighed the gain from the herd effects, as shown in Table 4. These key observations suggest that the marginal effect is more important than the herd effect for protection against COVID-19 mortality [16]. Thus, vaccination strategies should focus on how to capitalize on the marginal effect, i.e., promote individual vaccination willingness and accessibility, in order to continuously push for a higher vaccination rate in the population [16,34]. Based on our results, a vaccination campaign should be carefully balanced between pursuing higher vaccination coverage in the population and maintaining cost-effectiveness, and it should not solely aim at meeting a predefined threshold for triggering the herd effect [5,34,35].

Heterogeneity among the U.S. counties in terms of the marginal and herd effects is considerable. The sizes and even the signs of the marginal and herd effects could vary across all the counties, which indicates that the protection effect of COVID-19 vaccination is not constant and is partially determined by county idiosyncrasies. For example, we took the social vulnerability index (SVI) into account in our analysis and did find that the theme of household composition and disability was significantly associated with the CFR after controlling for the vaccination coverage. This indicates that the demographic features of individual county, such as the age distribution and disability proportion, play vital roles in explaining the heterogeneity that existed in the relationship between the vaccination coverage and CFR [36]. Although the other three SVI themes, namely, socioeconomic status, minority status and language, and housing type and transportation, were not statistically significant, factors such as environmental conditions [37], political atmosphere [38] and non-pharmaceutical interventions [39] could contribute to county heterogeneity, and potentially confound the relationship between the vaccination coverage and the CFR. Most notably, research has shown that vaccine hesitancy (willingness) is a key determinant of vaccination coverage, and it potentially mediates the relationship between the factors influencing the CFR (such as SVI) and the CFR itself, and, therefore, variation in vaccine hesitancy among the U.S. counties potentially accounts for a significant portion of the county heterogeneity observed in our paper [40,41].

There are limitations in our analysis: We did not investigate the impact of COVID-19 variants on the CFR and the vaccine effectiveness, considering there were different COVID-19 variants (and their lineages and sublineages), such as alpha, delta and omicron, spreading during our study period, as we could not clearly identify the boundaries of the spreading period of each variant from the data. For a similar reason, the potential impact of different brands of vaccines (such as BioNTech and Moderna) was also not considered in our model, as the data did not contain information about the number of administered doses of every specific brand. We also acknowledge that our model did not account for partial vaccination coverage or immunity gained from past infections, which could potentially bias the estimates of the herd and marginal effects. Reporting delays and errors may exist in the daily records of case fatality rates and vaccination coverages at county level, and, therefore, they became a source of potential bias for our analysis [39]. Notably, our model treats the breakpoints as the fixed values across all the counties, which may not be true as the breakpoints could vary across different counties as a result of unique evolvement of vaccination campaign in individual counties. Unfortunately, allowing each county to have its own breakpoints would require a huge number of parameters and a complex Bayesian model, which goes beyond the scope of this paper [42]. Most importantly, our findings about the relationship between vaccine coverage and CFR could be weakened by other confounding factors, such as the decreased pathogenicity of Omicron, existing health conditions and other possible immune evasions [43,44]. Therefore, further robustness and sensitivity analyses may be warranted to check the impact of potential confounders [45,46,47,48].

## 5. Conclusions

To summarize, we estimated the herd and marginal effects for reducing case fatality rate via a segmented regression model. Specifically, we identified three different breakpoints that represented the locations of the herd effects. Accounting for county heterogeneity, we found one of the three herd effects to be statistically significant, and this suggested that an additional indirect benefit of COVID-19 vaccination may exist at the earlier stage of a vaccination campaign. We also found that the marginal-effect size varied at different stages of the vaccination campaign, and, specifically, the marginal (direct) benefit of COVID-19 vaccination likely became larger as the vaccination coverage increased. Our findings demonstrate that the herd and marginal effects should be carefully differentiated and assessed in analyzing vaccination data, to better inform vaccination campaign strategies as well as evaluate vaccination effectiveness. Our study contributes to the existing literature on COVID-19 vaccination by confirming that vaccination against COVID-19 can reduce the mortality via both herd and marginal effects, though the reduced mortality can also be attributed to revolutionized care pathways for COVID-19 and more effective treatments [1]. We caution readers that our findings do not pre-empt the debates about whether rapid mass vaccination should be recommended for a highly mutating virus (such as COVID-19) and whether massive vaccination is more cost-effective than other preventive strategies.

## Figures and Tables

**Table 1:** The regression coefficient estimates for the analysis at national level. Their corresponding T-ratios and *p*-values are also provided.

Parameter	Estimate	T Ratio	*p*-Value
$\beta_{0}$	1.976	624.70	<0.001
$\beta_{1}$	0.000	0.28	0.78
$\alpha_{1}$	−0.003	−0.90	0.37
$\delta_{1}$	−0.057	−45.32	<0.001
$\alpha_{2}$	−0.236	−80.67	<0.001
$\delta_{2}$	0.003	7.50	<0.001
$\alpha_{3}$	−0.227	−55.09	<0.001
$\delta_{3}$	−0.115	−47.03	<0.001

**Table 2:** The herd- and marginal-effect estimates for the analysis at national level. Here, the marginal- and herd-effect estimates were calculated based on regression coefficients in Table 1, and the *p*-values of their T-tests were also obtained. The marginal-effect estimates were calculated for each local region $\Psi_{i}(i=1,2,3,4)$, which were separated by the breakpoints $b_{k}$, k=1,2,3.

Effect	Location	Estimate	*p*-Value
1st marginal effect	$\Psi_{1}=\left[8.66\%,32\%\right)$	0.000	0.78
1st herd effect	$b_{1}=32\%$	−0.003	0.37
2nd marginal effect	$\Psi_{2}=\left[32\%,36\%\right)$	−0.057	<0.001
2nd herd effect	$b_{2}=36\%$	−0.233	<0.001
3rd marginal effect	$\Psi_{3}=\left[36\%,47\%\right)$	0.003	<0.001
3rd herd effect	$b_{3}=47\%$	0.009	0.02
4th marginal effect	$\Psi_{4}=\left[47\%,49.28\%\right)$	−0.115	<0.001

**Table 3:** The mixed-model parameter estimates pertaining to fixed effects (F.E.) and the variances of their corresponding random effects (VRE) for the analysis at county level. Among the parameters, $\gamma_{1},\gamma_{2},\gamma_{3}$ and $\gamma_{4}$ characterize the first (socioeconomic status), second (household composition and disability), third (minority status and language) and fourth (housing type and transportation) CDC SVI theme rankings respectively.

Parameter	F.E. Est	F.E. T Ratio	F.E. *p*-Value	VRE Est
$\beta_{0}$	1.64	28.88	<0.001	1.33
$\beta_{1}$	−0.004	−8.85	<0.001	0.0005
$\alpha_{1}$	−0.025	−7.35	<0.001	0.024
$\delta_{1}$	−0.007	−3.74	<0.001	0.007
$\alpha_{2}$	−0.018	−2.84	0.005	0.091
$\delta_{2}$	−0.02	−13.07	<0.001	0.006
$\alpha_{3}$	−0.004	−0.35	0.728	0.2
$\delta_{3}$	−0.039	−23.49	<0.001	0.004
$\gamma_{1}$	0.0004	0.33	0.744	n/a ^1^
$\gamma_{2}$	0.807	11.00	<0.001	n/a
$\gamma_{3}$	−0.057	−0.73	0.467	n/a
$\gamma_{4}$	0.004	0.05	0.961	n/a

^1^ $\gamma_{1},\gamma_{2},\gamma_{3}$ and $\gamma_{4}$ did not have random effects as they were corresponded to the covariates at the county level.

**Table 4:** The herd- and marginal-effect estimates for the analysis at county level. The fixed-effect estimates (F.E. Est), the estimates of the variances of their corresponding random effects (VRE Est) as well as the *p*-value for the F.E Est, are provided.

Effect	Location	F.E. Est	F.E. *p*-Value	VRE Est
1st marginal effect	$\Psi_{1}=\left[8.66\%,32\%\right)$	−0.004	<0.001	0.0005
1st herd effect	$b_{1}=32\%$	−0.025	<0.001	0.024
2nd marginal effect	$\Psi_{2}=\left[32\%,36\%\right)$	−0.01	<0.001	0.008
2nd herd effect	$b_{2}=36\%$	0.008	0.70	0.115
3rd marginal effect	$\Psi_{3}=\left[36\%,47\%\right)$	−0.023	<0.001	0.006
3rd herd effect	$b_{3}=47\%$	0.014	0.545	0.29
4th marginal effect	$\Psi_{4}=\left[47\%,49.3\%\right)$	−0.043	<0.001	0.005

## Data Availability

Data is publicly available and can be found in the references. The specific dataset used in this project is available upon request.

## Supplementary Materials

The following supporting information can be downloaded at https://www.mdpi.com/article/10.3390/vaccines11061078/s1, File S1: Code and Raw Output.
