# Supplementary material for: Investigating the Marginal and Herd Effects of COVID-19 Vaccination for Reducing Case Fatality Rate: Evidence from the United States between March 2021 to January 2022
Source: Vaccines (Basel). 2023 Jun 9;11(6):1078. doi: 10.3390/vaccines11061078 (PMC10326830; doi:10.3390/vaccines11061078)
Supplement: Supplementary file 1 [file vaccines-11-01078-s001.zip › vaccines-2247996-supplementary.pdf]

1-R code:

```
## Read the vaccination dataset
vacc <- read.csv('COVID-19_Vaccinations_in_the_United_States_County.csv')
## See how the dataset looks like for a single county
v1 <- vacc[vacc$FIPS==21151,]
## Read the raw time-series case counts
case <- read.csv('Raw Data/time_series_covid19_confirmed_US.csv')
## Read the raw time-series death counts
death <- read.csv('Raw Data/time_series_covid19_deaths_US.csv')
## Creat case fatality ratio
cfr <- death[,-(1:12)]/case[,-(1:11)]
cfr <- cbind.data.frame(death[,1:12],cfr)
write.csv(cfr,'cfr.csv',row.names = FALSE)

## Code for cleaning the data should be put here.
## The cleaned data is project.csv
## The data project.csv needs to be further cleaned, to address the following issues:
## Get rid of unknown counties.
## Remove rows with missing values in ppct, sppct and cfr.

## Find the breakpoints
library(segmented)
library(tidyverse)
project <- read.csv('project.csv')
project <- project[,-6]
## project <- project[!is.na(project$FIPS),]
colnames(project) <-
c("date","fips","county","state","ppct","theme1","theme2","theme3","theme4","cfr")
## But we need to select the right period,i.e., between March 11, 2021 and Jan 26, 2022
## The kickoff date of vaccination campaign in the US is March 11, 2021
## The date President Biden announce all Americans are eligible for vaccination
## The end date is Jan 26, 2022 when CDC announce that more than 99.9% of the virus
are Omicron
project <-
project%>%mutate(date=as.Date(date),cfr=100*cfr)%>%filter(date>=as.Date("2021-3-
11")&date<=as.Date("2022-1-26"))
mod <- lm(cfr~ppct+theme1+theme2+theme3+theme4,data = project)
project1 <-
project%>%group_by(date)%>%summarise(mcfr=mean(cfr),mppct=mean(ppct))
plot(project1$mppct,project1$mcfr,type='l')

## First analysis
## Segmented regression based on the mean data project1
```

```
## National level
mod <- lm(mcfr~mppct,data=project1)
selgmented(mod,seg.Z = ~mppct,type = "bic",Kmax = 3)
smod <- segmented(mod,seg.Z = ~mppct,npsi = 3)

## We identified 3 breakpoints,i.e.,32,36 and 47
plot(project1$mppct,project1$mcfr,type='l')
abline(v=32,lty=2,col='red')
abline(v=36,lty=2,col='red')
abline(v=47,lty=2,col='red')

## Create figure 1
k=data.frame(mppct=c(32,36,47),mcfr=c(1.97,1.758,1.767))
project1%>%ggplot(aes(x=mppct/100,y=mcfr/100))+geom_line()+geom_point(data=k)
+scale_y_continuous(
name = "Mean CFR", labels = scales::label_percent())+scale_x_continuous(
  name = "Mean Vaccination Coverage", labels =
scales::label_percent())+geom_point(data = k)

## We created the segmented regression model ourselves
project1 <-
project1%>%mutate(d1=ifelse(mppct>=32&mppct<36,1,0),d2=ifelse(mppct>=36&mp
pct<47,1,0),d3=ifelse(mppct>=47,1,0))
project1 <- project1%>%mutate(mppct1=d1*(mppct-32),mppct2=d2*(mppct-
36),mppct3=d3*(mppct-47))
mod1 <- lm(mcfr~mppct+d1+d2+d3+mppct1+mppct2+mppct3,data = project1)
summary(mod1)

## Calculate the p-values associated with the herd and marginal effects
m=vcov(mod1)
coe=coef(mod1)
## herd2
v=c(0,0,-1,1,0,0,0,0)
2*pt(sum(coe*v)/sqrt(t(v)%*%m%*%v),314)
## herd3
v=c(0,0,0,-1,1,0,0,0)
2*(1-pt(sum(coe*v)/sqrt(t(v)%*%m%*%v),314))
## margin2
v=c(0,1,0,0,0,1,0,0)
2*pt(sum(coe*v)/sqrt(t(v)%*%m%*%v),314)
## margin3
v=c(0,1,0,0,0,0,1,0)
2*(1-pt(sum(coe*v)/sqrt(t(v)%*%m%*%v),314))
## margin4
```

```
v=c(0,1,0,0,0,0,0,1)
2*pt(sum(coe*v)/sqrt(t(v)%*%m%*%v),314)

## Run mixed models
## First create dummy variables based on the three breakpoints
project <-
project%>%mutate(d1=ifelse(ppct>=32&ppct<36,1,0),d2=ifelse(ppct>=36&ppct<47,1,
0),d3=ifelse(ppct>=47,1,0))
project <- project%>%mutate(ppct1=d1*(ppct-32),ppct2=d2*(ppct-
36),ppct3=d3*(ppct-47))
## See an example
dat <- project%>%filter(fips==6113)
mod <- lm(cfr~ppct+d1+d2+d3+ppct1+ppct2+ppct3,data = dat)
summary(mod)
plot(dat$ppct,dat$cfr,type='l')

## Use lmer function
library(lme4)
library(RLRsim)
mixmod0 <-
lmer(cfr~1+ppct+d1+d2+d3+ppct1+ppct2+ppct3+(1|fips)+theme1+theme2+theme3
+theme4,data =
project,REML=FALSE,control=lmerControl(optimizer="bobyqa",calc.derivs =
FALSE,optCtrl=list(maxfun=2e5)))
mixmod1 <-
lmer(cfr~1+ppct+d1+d2+d3+ppct1+ppct2+ppct3+(1+ppct+ppct1+ppct2+ppct3|fips)
+theme1+theme2+theme3+theme4,data =
project,REML=FALSE,control=lmerControl(optimizer="bobyqa",calc.derivs =
FALSE,optCtrl=list(maxfun=2e5)))
mixmod2 <-
lmer(cfr~1+ppct+d1+d2+d3+ppct1+ppct2+ppct3+(1+ppct+d1+d2+d3+ppct1+ppct2
+ppct3|fips)+theme1+theme2+theme3+theme4,data =
project,REML=FALSE,control=lmerControl(optimizer="bobyqa",calc.derivs =
FALSE,optCtrl=list(maxfun=2e5)))

## Running the code above (about mixed models) was quite slow in R.
## So we used STATA to run the same models, see details in the appendix.

## Calculate the p-values associated with the herd and marginal effects
## herd2
tst=(-0.0175721+0.0251904)/(0.00001174+0.0003834-2*6.209e-07)^0.5
2*(1-pt(tst,310))
## herd3
tst=(-0.0039231+0.0175721)/(0.0001276+0.0003834-2*2.033e-06)^0.5
```

```
2*(1-pt(tst,310))
## margin2
tst=(-0.0036004-0.0067726)/(1.654e-07+3.284e-06-2*4.495e-09)^0.5
2*pt(tst,310)
## margin3
tst=(-0.0036004-0.0196573)/(1.654e-07+2.261e-06-2*4.983e-09)^0.5
2*pt(tst,310)
## margin4
tst=(-0.0036004-0.0393751)/(1.654e-07+2.809e-06-2*5.985e-09)^0.5
2*pt(tst,310)
```

2-Analysis at county level (mixed model) using STATA (the maximum likelihood estimation method was used for implementing all the models below):

1. model1: random effects for all terms except theme1 through theme4

```
. mixed c.cfr c.vc i.d1 i.d2 i.d3 c.vc1 c.vc2 c.vc3 theme1 theme2 theme3 theme4 || fips: c.vc i.d1 i.d2 i.d3 c
> .vc1 c.vc2 c.vc3, mle
```

Output:

```
Mixed-effects ML regression                     Number of obs     =  1,001,098
Group variable: fips                            Number of groups  =      3,109
                                                Obs per group:
                                                              min =        322
                                                              avg =      322.0
                                                              max =        322
                                                Wald chi2(11)     =    1016.31
Log likelihood =  513590.06                     Prob > chi2       =     0.0000
```

cfr	Coefficient	Std. err.	z	P>|z|	[95% conf.	interval]
vc	-.0036004	.0004067	-8.85	0.000	-.0043975	-.0028034
1.d1	-.0251904	.0034266	-7.35	0.000	-.0319065	-.0184743
1.d2	-.0175721	.0061915	-2.84	0.005	-.0297073	-.0054369
1.d3	-.0039231	.0112962	-0.35	0.728	-.0260633	.0182171
vc1	-.0067726	.0018122	-3.74	0.000	-.0103244	-.0032208
vc2	-.0196573	.0015037	-13.07	0.000	-.0226045	-.01671
vc3	-.0393751	.0016761	-23.49	0.000	-.0426603	-.0360899
theme1	.0003766	.0011555	0.33	0.744	-.0018882	.0026414
theme2	.8068916	.0733619	11.00	0.000	.6631049	.9506783
theme3	-.0573455	.0788306	-0.73	0.467	-.2118507	.0971597
theme4	.0038997	.0806247	0.05	0.961	-.1541218	.1619213
_cons	1.640176	.0567852	28.88	0.000	1.528879	1.751473

Random-effects parameters	Estimate	Std. err.	[95% conf.	interval]
fips: Independent				
var(vc)	.0004989	.0000129	.0004743	.0005248
var(1.d1)	.0238676	.0007884	.0223713	.0254639
var(1.d2)	.0912826	.0028374	.0858875	.0970167
var(1.d3)	.1991367	.0074995	.1849673	.2143915
var(vc1)	.0072339	.000239	.0067802	.0077178
var(vc2)	.0056307	.0001856	.0052784	.0060064
var(vc3)	.0042457	.0001783	.0039102	.0046099
var(_cons)	1.32611	.0338076	1.261477	1.394055
var(Residual)	.0189058	.000027	.0188529	.0189588

```
LR test vs. linear model: chi2(8) = 3.6e+06                 Prob > chi2 = 0.0000

Note: LR test is conservative and provided only for reference.
```

The covariance of random effects

```
. estat recovariance

Random-effects covariance matrix for level fips
```

	d1	d2	d3	ppct	ppct1	ppct2
d1	.0238676					
d2	0	.0912826				
d3	0	0	.1991367			
ppct	0	0	0	.0004989		
ppct1	0	0	0	0	.0072339	
ppct2	0	0	0	0	0	.0056307
ppct3	0	0	0	0	0	0
_cons	0	0	0	0	0	0

	ppct3	_cons
ppct3	.0042457	
_cons	0	1.32611

The covariance matrix of the fixed effects in model 1:

```
. estat vce

Covariance matrix of coefficients of mixed model
```

e(V)	cfr<br>vc	1.<br>d1	1.<br>d2	1.<br>d3	vc1	vc2	vc3
**cfr**							
vc	1.654e-07						
1.d1	-2.852e-08	.00001174					
1.d2	-7.419e-08	6.209e-07	.00003834				
1.d3	-1.566e-07	1.011e-06	2.033e-06	.0001276			
vc1	-4.495e-09	-7.162e-07	9.894e-08	1.485e-07	3.284e-06		
vc2	-4.983e-09	2.775e-08	-2.885e-07	1.843e-07	4.746e-09	2.261e-06	
vc3	-5.985e-09	3.483e-08	6.949e-08	-1.201e-07	5.876e-09	6.619e-09	2.809e-06

Save this model as m1 in STATA.

```
. est sto m1
```

2. We noticed that the random effect sizes of vc, vc1, vc2, vc3 were small, and therefore we considered removing the random effects of those four terms in the model2.

```
. mixed c.cfr c.vc i.d1 i.d2 i.d3 c.vc1 c.vc2 c.vc3 theme1 theme2 theme3 theme4
> || fips: i.d1 i.d2 i.d3, mle
```

Output:

```
Iteration 0:   log likelihood =   209406.2
Iteration 1:   log likelihood =   209406.2

Computing standard errors ...

Mixed-effects ML regression                     Number of obs     =  1,001,098
Group variable: fips                            Number of groups  =      3,109
                                                Obs per group:
                                                              min =        322
                                                              avg =      322.0
                                                              max =        322
                                                Wald chi2(11)     =   84270.90
Log likelihood =   209406.2                     Prob > chi2       =     0.0000
```

cfr	Coefficient	Std. err.	z	P>|z|	[95% conf.	interval]
vc	-.0058018	.0000486	-119.40	0.000	-.005897	-.0057065
1.d1	.0042839	.0056506	0.76	0.448	-.0067912	.015359
1.d2	.0395135	.0067861	5.82	0.000	.0262129	.052814
1.d3	.0471648	.0103839	4.54	0.000	.0268126	.0675169
vc1	-.0037126	.0005969	-6.22	0.000	-.0048825	-.0025427
vc2	-.0112739	.0001549	-72.77	0.000	-.0115776	-.0109703
vc3	-.0150956	.0001016	-148.56	0.000	-.0152948	-.0148965
theme1	.0002682	.0009363	0.29	0.775	-.001567	.0021034
theme2	.882877	.059442	14.85	0.000	.7663728	.9993812
theme3	-.0280334	.0639139	-0.44	0.661	-.1533022	.0972355
theme4	-.0121168	.0653133	-0.19	0.853	-.1401285	.1158948
_cons	1.643514	.0460278	35.71	0.000	1.553301	1.733727

Random-effects parameters	Estimate	Std. err.	[95% conf.	interval]
fips: Independent				
var(1.d1)	.0751658	.0021548	.0710589	.0795101
var(1.d2)	.1188571	.0032836	.1125925	.1254703
var(1.d3)	.1795017	.0062097	.1677343	.1920947
var(_cons)	.8738916	.0221827	.8314781	.9184686
var(Residual)	.0362979	.0000516	.036197	.0363992

```
LR test vs. linear model: chi2(4) = 3.0e+06               Prob > chi2 = 0.0000

Note: LR test is conservative and provided only for reference.
```

Save this model as m2 in STATA.

```
. est sto m2
```

3. Using Likelihood Ratio Test to compare m1 and m2.

```
. lrtest m1 m2, stats

Likelihood-ratio test
Assumption: m2 nested within m1

 LR chi2(4) = 608367.73
Prob > chi2 =    0.0000

Note: The reported degrees of freedom assumes the null hypothesis is not on
      the boundary of the parameter space. If this is not true, then the
      reported test is conservative.

Akaike's information criterion and Bayesian information criterion
```

Model	N	ll(null)	ll(model)	df	AIC	BIC
m2	1,001,098	.	209406.2	17	-418778.4	-418577.5
m1	1,001,098	.	513590.1	21	-1027138	-1026890

```
Note: BIC uses N = number of observations. See [R] BIC note.
```

We rejected the null hypothesis and concluded that m1 was better than m2.

4. **Furthermore, we observed that the random effect size of vc was the smallest. Therefore, we only removed the random effect of vc in the model3 and save this model as m3 in STATA.**

```
Performing EM optimization ...

Performing gradient-based optimization:
Iteration 0:   log likelihood =  322562.17
Iteration 1:   log likelihood =  322562.17

Computing standard errors ...

Mixed-effects ML regression                     Number of obs     =  1,001,098
Group variable: fips                            Number of groups  =      3,109
                                                Obs per group:
                                                              min =        322
                                                              avg =      322.0
                                                              max =        322
                                                Wald chi2(11)     =   19757.01
Log likelihood =  322562.17                     Prob > chi2       =     0.0000

------------------------------------------------------------------------------
         cfr | Coefficient  Std. err.      z    P>|z|     [95% conf. interval]
-------------+----------------------------------------------------------------
          vc |  -.0057919   .0000429  -135.01   0.000     -.005876   -.0057078
        1.d1 |   .0003946   .0047291     0.08   0.934    -.0088743    .0096634
        1.d2 |   .0289429   .0066543     4.35   0.000     .0159007    .0419852
        1.d3 |   .0863778   .0097953     8.82   0.000     .0671793    .1055763
         vc1 |  -.0045298   .0018739    -2.42   0.016    -.0082025   -.0008571
         vc2 |  -.0151826   .0013547   -11.21   0.000    -.0178378   -.0125274
         vc3 |  -.0336915   .0015201   -22.16   0.000    -.0366709    -.030712
      theme1 |   .0002685   .0009378     0.29   0.775    -.0015696    .0021066
      theme2 |   .8856445   .0595413    14.87   0.000     .7689456    1.002343
      theme3 |  -.0279839   .0640191    -0.44   0.662     -.153459    .0974912
      theme4 |  -.0143341   .0654228    -0.22   0.827    -.1425604    .1138923
       _cons |   1.642384   .0461006    35.63   0.000     1.552028     1.73274
------------------------------------------------------------------------------

------------------------------------------------------------------------------
  Random-effects parameters  |   Estimate   Std. err.     [95% conf. interval]
-----------------------------+------------------------------------------------
fips: Independent            |
                   var(1.d1) |   .0490266   .0014968      .0461791    .0520498
                   var(1.d2) |   .1100243   .0032219      .1038872     .116524
                   var(1.d3) |   .1573158   .0056196      .1466784    .1687247
                    var(vc1) |   .0073673    .000241      .0069097    .0078551
                    var(vc2) |   .0044818   .0001504      .0041965    .0047865
                    var(vc3) |   .0034149   .0001469      .0031388    .0037152
                  var(_cons) |   .8768629   .0222595      .8343026    .9215942
-----------------------------+------------------------------------------------
               var(Residual) |   .0282662   .0000403      .0281873    .0283453
------------------------------------------------------------------------------
LR test vs. linear model: chi2(7) = 3.2e+06               Prob > chi2 = 0.0000

Note: LR test is conservative and provided only for reference.

.  est sto m3
```

5. Use Likelihood Ratio Test to compare m1 and m3.

```
. lrtest m1 m3, stats

Likelihood-ratio test
Assumption: m3 nested within m1

 LR chi2(1) = 382055.78
Prob > chi2 =    0.0000

Note: The reported degrees of freedom assumes the null hypothesis is not on the boundary of the parameter
      space. If this is not true, then the reported test is conservative.

Akaike's information criterion and Bayesian information criterion
```

Model	N	ll(null)	ll(model)	df	AIC	BIC
m3	1,001,098	.	322562.2	20	-645084.3	-644848
m1	1,001,098	.	513590.1	21	-1027138	-1026890

```
Note: BIC uses N = number of observations. See [R] BIC note.
```

We rejected the null hypothesis and concluded that m1 was still better than m3.
